# Expanded host and geographic range of tadpole associations with the Severe Perkinsea Infection group

**DOI:** 10.1098/rsbl.2021.0166

**Published:** 2021-06-16

**Authors:** Vanessa Smilansky, Miloslav Jirků, David S. Milner, Roberto Ibáñez, Brian Gratwicke, Andrew Nicholls, Julius Lukeš, Aurélie Chambouvet, Thomas A. Richards

**Affiliations:** ^1^ Living Systems Institute and Biosciences, University of Exeter, Exeter, Devon EX4 4QD, UK; ^2^ Institute of Parasitology, Biology Centre, Czech Academy of Sciences, 370 05 České Budějovice, Czech Republic; ^3^ Department of Zoology, University of Oxford, Oxford OX1 3SZ, UK; ^4^ Smithsonian Tropical Research Institute, Panamá, Republic of Panama; ^5^ Sistema Nacional de Investigación, SENACYT, Panamá, Republic of Panama; ^6^ Smithsonian National Zoo and Conservation Biology Institute, Washington D.C., USA; ^7^ Faculty of Sciences, University of South Bohemia, 370 05 České Budějovice, Czech Republic; ^8^ CNRS, Université of Brest, IRD, Ifremer, LEMAR, Plouzané, France

**Keywords:** frog disease, amphibian conservation, alveolate parasites

## Abstract

Severe Perkinsea infection is an emerging disease of amphibians, specifically tadpoles. Disease presentation correlates with liver infections of a subclade of Perkinsea (Alveolata) protists, named Pathogenic Perkinsea Clade (PPC). Tadpole mortality events associated with PPC infections have been reported across North America, from Alaska to Florida. Here, we investigate the geographic and host range of PPC associations in seemingly healthy tadpoles sampled from Panama, a biogeographic provenance critically affected by amphibian decline. To complement this work, we also investigate a mortality event among *Hyla arborea* tadpoles in captive-bred UK specimens. PPC SSU rDNA was detected in 10 of 81 Panama tadpoles tested, and *H. arborea* tadpoles from the UK. Phylogenies of the Perkinsea SSU rDNA sequences demonstrate they are highly similar to PPC sequences sampled from mortality events in the USA, and phylogenetic analysis of tadpole mitochondrial SSU rDNA demonstrates, for the first time, PPC associations in diverse hylids. These data provide further understanding of the biogeography and host range of this putative pathogenic group, factors likely to be important for conservation planning.

## 1. Introduction

Severe Perkinsea infection (SPI) has been associated with tadpole mass mortality events (MMEs) in the USA [1] and likely represents the third most common infectious disease of amphibians in North America [1]. The disease pathology has been associated with a specific group of Perkinsea protists (syn. Perkinsids, Perkinsozoa) called Pathogenic Perkinsea Clade (PPC) based on small-subunit ribosomal DNA (SSU rDNA) sequencing [1,2]. This group is part of a wider clade of the freshwater Perkinsea named Novel-Alveolate-Group-01 (NAG01) [3]. Tadpole associations with specific clades of NAG01-Perkinsea have been detected across multiple continents [3], yet the PPC clade associated with SPI has only been detected in the USA, and mainly from SPI-diseased tadpoles [1,2]. Formal identification of the disease relationship satisfying Koch's postulates is absent [4]. Yet, in localities with SPI outbreaks, one study demonstrated that 100% of tadpoles exhibiting SPI were PPC-positive, whereas a cohort of asymptomatic tadpoles sampled nearby demonstrated only 2.5% (2/81) PPC prevalence [1]. This pattern provides circumstantial evidence for a relationship between the disease (SPI) and the protist infection (PPC) [5,6].

The first documented MMEs associated with SPI were recorded in New Hampshire in 1999 [7] and, since then, SPI-associated mortality events have been reported in seven other states across a broad geographic range [1,2,7,8]. Most of these SPI-associated MMEs have occurred in *Lithobates* spp. tadpoles [1,2,8,9], but have also been detected in larval hylids (Hylidae), including *Pseudacris crucifer* (spring peeper) and *Acris gryllus* (southern cricket frog) [1]. Here, we investigate the wider geographic and host range of PPC by screening tadpoles from Panama and a candidate SPI outbreak in UK captive-bred specimens. Using SSU rDNA sequencing, we demonstrate a hitherto unsampled biogeographic and host-associated taxonomic provenance of this putative pathogen, suggesting that PPC may be more biogeographically widespread than previously assumed, a finding that is crucial in managing animal trade and directing future conservation efforts.

## 2. Material and methods

### (a) Sample collection and preparation

Fieldwork was conducted in Panama (November 2018). Eighty-one tadpoles, without apparent pathologies and with unknown species identification, were collected from seven sites (electronic supplementary material, tables S1 and S2). New dissection consumables were used for processing each distinct sample set and flame-sterilized between processing each individual tadpole; each tadpole was individually washed in H_2_O, euthanized using MS-222 and washed in physiological saline before dissection. Liver tissues were stored in LifeGuard™ (Qiagen) at −20°C for 1–2 weeks and then at −80°C. All livers appeared normal upon gross visual inspection. Due to permit regulations, all DNA samples were extracted in Panama.

Captive-bred *Hyla arborea* tadpoles were collected from a single-aquarium MME (AME1) in Surrey, UK, in July 2019 (electronic supplementary material, table S1) and stored in LifeGuard™ at −80°C. All the livers from tadpoles collected in the UK showed signs of SPI, specifically enlargement and yellow discoloration (identified using a dissecting microscope at 10× magnification). Subsamples of UK tadpoles were preserved for histopathological microscopy (electronic supplementary material, figure S1). Due to the small size of these tadpoles, two groups of five dissected livers from the same MME were pooled for DNA analysis.

DNA was extracted using the DNeasy Blood and Tissue Kit (Qiagen) with an overnight lysis at 37°C and disruption with 425–600 µm acid-washed glass beads (Sigma-Aldrich) on a FastPrep-24™ tissue homogenizer (MP Biomedicals). Each batch of DNA extractions included a ‘blank’ replicate, processed in the same manner as the tissue samples, but without tissue samples. New sterile tools, collection tubes and gloves were used between each sample recovery and individual sample preparation. However, we cannot exclude the possibility that positive PPC detection may be due to contamination from the surface of the tadpole and not directly from the liver tissue, specifically in the case of the tadpoles sampled in Panama. Indeed, such errors can arise in PCR screens [10]. Nonetheless, such a result would still infer that a putative parasite within the SPI-associated PPC-group was in close environmental association with the tadpoles recovered.

### (b) NAG01 screening

Two hundred and ninety base pairs of SSU rDNA sequence was amplified using 300F-B [3] and NAG01R_1 primers (electronic supplementary material, table S3). Each 25 µl reaction comprised 1× PCR Master Mix (Promega, containing *Taq* DNA polymerase), 500 nM of each primer and 5 µl DNA. Each assay included a negative (no-template) and positive control (1 µl of DNA from PPC-infected *L. sylvaticus* tadpole liver (KNA_DNA [11])). Extraction blanks (5 µl) were screened alongside liver DNA samples; these extraction controls were all negative. Cycling conditions were 2 min at 95°C, followed by 35 cycles of 30 s at 95°C, 30 s at 56°C and 60 s at 72°C, with an additional 10 min 72°C extension. PCR products were checked on a 2% agarose gel and purified using the GeneJET PCR Purification Kit (Thermo Fisher).

PCR products were cloned using the StrataClone PCR Cloning Kit (Agilent Technologies) following the manufacturer's protocol. Plasmid DNA was extracted from transformants using the GeneJET Plasmid Miniprep Kit (Thermo Fisher) and Sanger sequenced externally using standard T3- and T7-primers (Eurofins Genomics). Contigs were assembled using Sequencher (v. 5.4.6). To determine if the DNA samples contained additional NAG01 diversity, we triplicated the PCR, cloning and sequencing (electronic supplementary material, table S4). One of these samples (UK_HA01-5) was subjected to a second triplicate PCR with a lower annealing temperature (54°C) to further test for clade diversity by decreasing primer specificity.

### (c) PPC SSU rDNA phylogeny

The sequences (electronic supplementary material, table S4) were assembled with representative sequences from the NCBI ‘nr’ database (August 2019) and automatically aligned in SeaView (v. 4.6.2) [12] (alignment: [13]). The alignment was adjusted manually to maximize accuracy and masked to remove gap-rich and poorly aligned sites. Maximum-likelihood (ML) and Log-Det distance [14] neighbour joining [15] (a partial correction for compositional bias [16]) were performed using IQ-TREE (v. 1.6.5) [17]. For ML analysis, a TIM3e + G4 model was used (identified by ModelFinder [18]). For both analyses, 1000 bootstrap replicates were performed.

### (d) Host identification

A long-term goal of this research is to develop protocols for large-scale screening technologies to facilitate amphibian conservation, preferably using at-bench and/or in-the-field technologies (e.g. Nanopore sequencing). As such, we adapted and tested a frog rDNA taxonomic barcode sequencing protocol for parallel-MinION™ amplicon sequencing: see electronic supplementary material, table S1 legend for methodological details. This approach allowed rapid identification of tadpole taxonomy, allowing tadpole collections to remain within permit limits, and without the need to export biological materials, or for complex/cumbersome laboratory-based instrumentation [19].

### (e) Host species phylogeny

The amphibian mitochondrial rDNA sequences were searched against the NCBI ‘nr’ database using BLASTn (June-2019), allowing preliminary taxonomic identification. The sequences were aligned using the methodology described above (alignment: [13]). For the ML analysis, a GTR + F + I + G4 model was selected.

## 3. Results

Liver tissues from 91 tadpoles (81 from Panama and 10 from the UK) were screened for NAG01-Perkinsea (electronic supplementary material, table S1). NAG01-Perkinsea SSU rDNA was detected in 10 Panama samples (all single tadpole specimens), and one pooled sample of five ‘tiny’ tadpoles from the same UK aquarium source. We recovered 1–10 clones for each sample (electronic supplementary material, table S4) and replicated the PCR and cloning steps for three samples for additional sampling (two from Panama: PA_T135, PA_T139, and one from the UK: UK_HA01-5), resulting in 72 sequences (electronic supplementary material, table S4).

**Figure 1.**
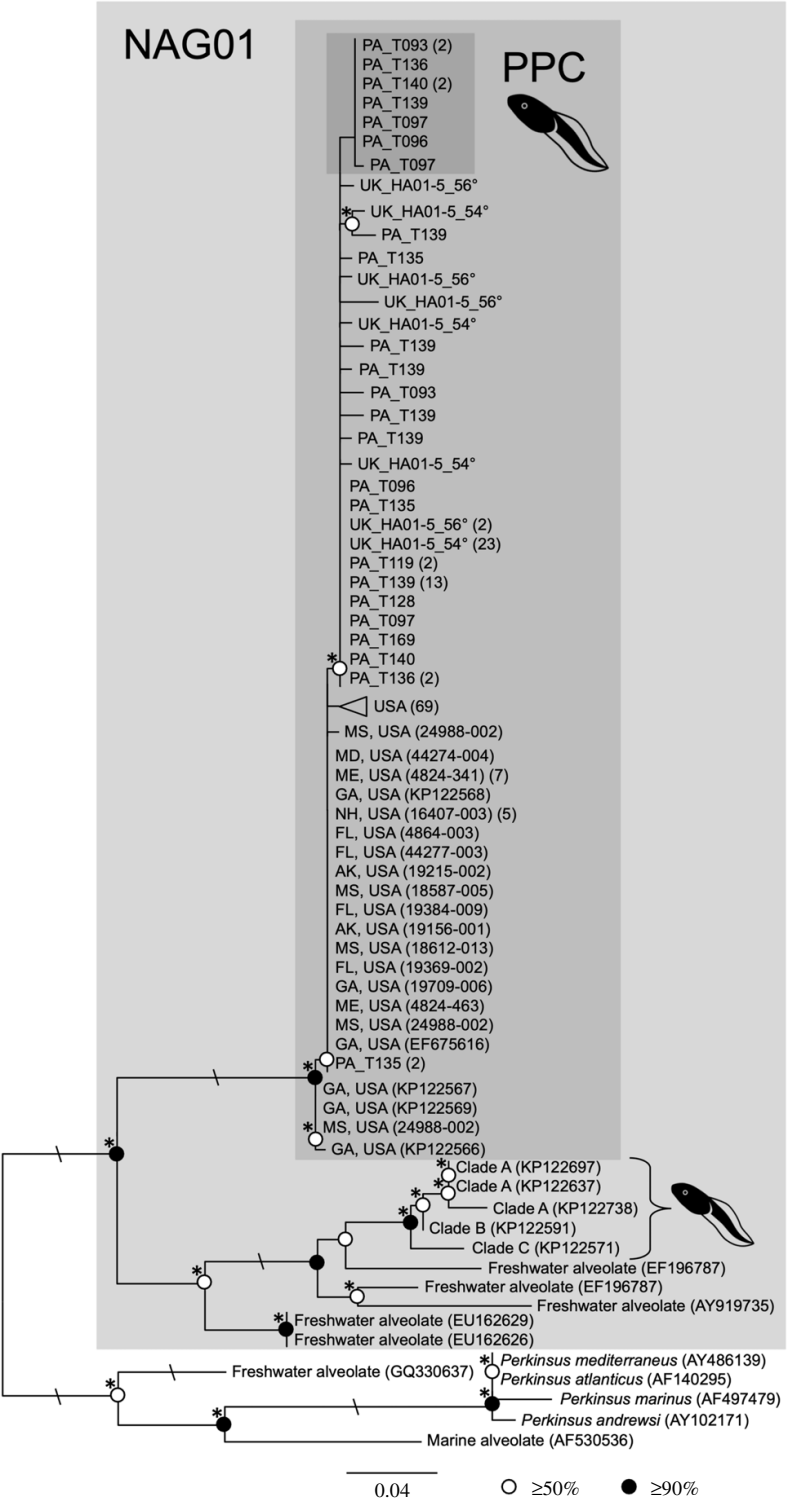
ML phylogeny of PPC SSU rDNA calculated from an alignment of 188 sequences and 288 sites. Bootstrap values are summarized: black circles = node support ≥90%; white circles = node support ≥50%. Asterisks (*) indicates node consistent with the Log-Det neighbour-joining bootstrap analysis at greater than or equal to 50%. Slashes (\) indicate branch lengths reduced by half. Newly generated PPC sequences are labelled with location (PA or UK) and sample ID. The Panama specific subgroup is highlighted by a dark grey box. Reference PPC sequences are labelled with geographic location and GenBank accession number or sample ID (this study). The triangle represents a subset of reference USA-PPC sequences; number of sequences is listed in parentheses. All other parenthesized numbers correspond to the number of sequences represented by each phylotype. Accession numbers for the newly generated sequences are provided in electronic supplementary material, table S4. For Mase format alignment see [13].

The 72 NAG01-Perkinsea SSU rDNA sequences generated from both the Panama and the UK samples were highly similar to PPC sequences from the USA [1,2]. Two sequences from PA_T135 grouped with a subset of USA-derived sequences (figure 1). To exclude the possibility that the UK-Panama similarities were the product of contamination, we replicated a subset of the PCR and cloning steps, confirming this shared rDNA-diversity. On all occasions we conducted PCR, numerous subsets of tadpole samples, the negative PCR controls (molecular-grade H_2_O), and the DNA extraction controls were amplicon negative.

A subset of the UK samples preserved in LifeGuard™ solution was sectioned for histopathology. The subsequent micrographs demonstrated evidence of candidate Perkinsea cells (electronic supplementary material, figure S1) similar to other histopathological observations of SPI [2].

**Figure 2.**
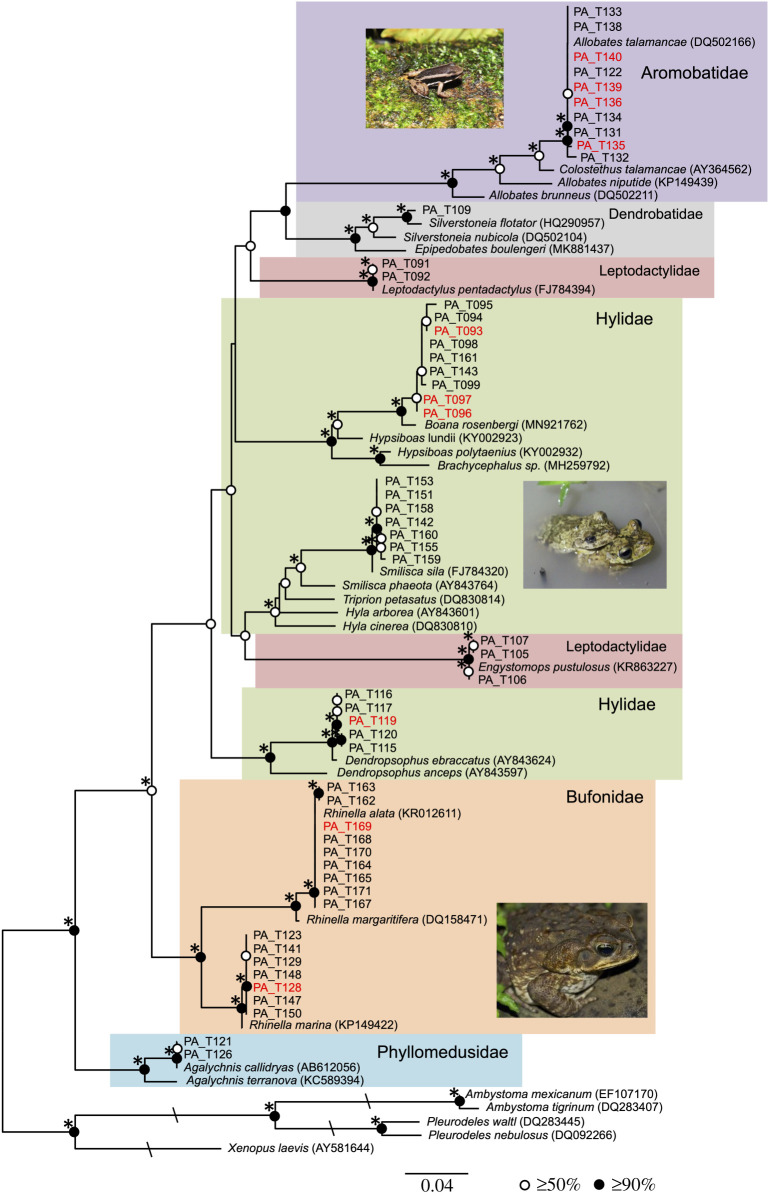
ML phylogeny of host mitochondrial rDNA sequences from the Panama tadpoles calculated from an alignment of 85 sequences and 410 sites. Bootstrap and node and Log-Det node support values are summarized as in figure 1. Samples with evidence for PPC associations are coloured red. Please note taxonomic names are derived from GenBank and are likely to be amended under recent taxonomic revisions, see electronic supplementary material, table S1 for contemporaneous updates. The tree includes *H. arborea* shown to be subject to repeat MMEs in UK aquaculture. Photographs depict *Rhinella marina* currently named *Rhinella horribilis* (Bufonidae) for Panamanian specimens, *Allobates talamancae* (Aromobatidae) (Credit: P. Kirillov License: https://creativecommons.org/licenses/by-sa/2.0/deed.en), and *Boana rosenbergi* (Hylidae). For Mase format alignment see [13].

Phylogenetic analysis revealed that the 55 newly generated Panama tadpole mitochondrial rDNA sequences represented six anuran families: Bufonidae, Phyllomedusidae, Aromobatidae, Hylidae, Leptodactylidae and Dendrobatidae (figure 2). The 10 PPC-positive Panama samples were grouped in strongly supported clades and so were shown to be present in Bufonidae (*n* = 2), Aromobatidae (*n* = 4), and Hylidae (*n* = 4) (figure 2).

## 4. Discussion

The aim of this study was to investigate if PPC–tadpole associations had a biogeographic range beyond North America. We surveyed tadpoles from natural populations in Panama, a geographic region subject to extensive amphibian decline [20], and investigated a disease outbreak in a UK captive population. These results revealed evidence for PPC–tadpole associations in both regions, and generated PPC sequences identical, or nearly identical, to published PPC sequences from USA MMEs. These data demonstrate that PPC host range is broader than previously indicated [1,2], as the PPC-positive samples include Hyloidea species, including two from the Bufonidae family (PA_T128 and PA_T169 - figure 2), one of the most widespread and speciose anuran families [21]. Given the association between PPC and MMEs [1], the identification of PPC in additional locales, and diverse hosts, this result has potentially important implications for global frog conservation.

Phylogenetic analysis identified additional diversity within the PPC clade which could be a product of DNA polymerase errors, strain variation or intranuclear variation (figure 1, alignment available at [13]), with the latter known to be a factor in many alveolates [22]. However, the sequences show little geographical variation, suggesting: (i) the rDNA sequence region sampled is not subject to sufficiently high rates of mutation to allow identification of biogeographical structure, and/or (ii) the PPC clade has rapidly spread between the USA, UK and Panama. The latter possibility would have significant conservation implications, discussed below. However, at this point, we note that the results presented in figure 1 do not allow us to identify either strain/species or biogeographical phylogenetic structure and, as such, the phylogeny serves only to confirm that all sequences detected are part of a closely related clade within the SPI/PPC radiation. Further biogeographic and strain phylogenetic structure could potentially be resolved by sequencing additional, rapidly evolving markers, allowing further insights into the spread of PPC and host/microbe coevolution, as has been investigated for marine Perkinsea parasites [23,24].

Previously published North American PCR surveys found cryptic PPC associations without evidence of disease to be rare; indeed, only two out of 81 tadpoles that appeared asymptomatic (*L. sphenocephalus*) were PCR-positive for PPC [1]. Our results show that cryptic PPC associations without evidence for the disease are more prevalent than previously indicated, as all 10 of the PPC-positive Panama specimens appeared normal upon gross examination, demonstrating that PPC is present in Panama, but with no evidence of a disease-causing association. Interestingly, most of the SPI-associated tadpole MMEs in North America have been documented in ranids, i.e. superfamily Ranoidea [1–3,7–9], whereas the PPC-positive samples from this study were all Hyloidea. This disparity suggests that Hyloidea spp. may be more tolerant of infection. However, we note that the PPC-positive DNA samples from the UK were derived from symptomatic tissue from *H. arborea* (Hyloidea), and the SPI disease phenotype has been reported in two North American hylid species, *Acris gryllus* and *Pseudacris crucifer* [1]. Alternatively, the disparity observed might simply reflect the larger ratio of hylids-to-ranids in Central America compared to North America [25], or could be a product of differing time frames of coevolutionary interaction in different geographic areas, an important factor when considering recent candidate parasite introductions.

The discovery of PPC in UK captive populations raises concerns regarding PPC transmission. It is not possible to track the origin of the infection; the *H. arborea* frogs are bred separately from other amphibians, and the disease-causing agent could have been introduced from adult *H*. *arborea* frogs from a UK outlet in 2018, or by unidentified tank water or equipment contamination. Aquaculture disease outbreaks pose a threat to native species [26], as captive-bred/farmed animals (and their pathogens) often spread from aquaculture to natural environments [27]. Currently, we are not aware of any other PPC-linked MMEs in wild amphibian populations in the UK or elsewhere in Europe, but further research is necessary. Pathogens can be unintentionally spread across a large geographical range when captive-bred amphibians are translocated for trade, food and other commercial purposes; a phenomenon known as ‘pathogen pollution’ [28,29]. This has alarming implications for conservation, as many amphibian species are currently suffering catastrophic population decline [30]. Central America is home to a large diversity of amphibian species, many of which are in decline [31] and some now persist in local captive assurance colonies [32]. Our survey for NAG01-Perkinsea associations in Panama tadpoles did not yield evidence of SPI-associated disease. However, our detection of PPC DNA associated with a diverse group of amphibian hosts in Central America is a cause for concern. In the light of the current imperilled state of Panama amphibians, and the threat that infectious disease outbreaks pose to captive assurance colonies [33], our findings encourage management strategies, including routine monitoring for PPC.

## Data accessibility

Alignments for PPC small-subunit rDNA and amphibian mitochondrial rDNA sequences are available at https://doi.org/10.6084/m9.figshare.13108136.v1. Additional datasets supporting this article have been uploaded as part of the electronic supplementary material. The data are provided in the electronic supplementary material [34].

## References

[1] Isidoro-Ayza M, Lorch JM, Grear DA, Winzeler M, Calhoun DL, Barichivich WJ. 2017 Pathogenic lineage of Perkinsea associated with mass mortality of frogs across the United States. Sci. Rep. **7**, 10288. (10.1038/s41598-017-10456-1) 28860470 PMC5579288

[2] Davis AK, Yabsley MJ, Kevin Keel M, Maerz JC. 2007 Discovery of a novel alveolate pathogen affecting southern leopard frogs in Georgia: description of the disease and host effects. EcoHealth **4**, 310-317. (10.1007/s10393-007-0115-3)

[3] Chambouvet A et al. 2015 Cryptic infection of a broad taxonomic and geographic diversity of tadpoles by Perkinsea protists. Proc. Natl Acad. Sci. USA **112**, E4743. (10.1073/pnas.1500163112) 26261337 PMC4553764

[4] Koch R. 1884 The etiology of tuberculosis. Germ. Theory Dis. **2**, 1-88.

[5] Chambouvet A et al. 2020 Diverse alveolate infections of tadpoles, a new threat to frogs? PLoS Pathog. **16**, e1008107. (10.1371/journal.ppat.1008107) 32053700 PMC7017987

[6] Fredricks DN, Relman DA. 1996 Sequence-based identification of microbial pathogens: a reconsideration of Koch's postulates. Clin. Microbiol. Rev. **9**, 18-33. (10.1128/CMR.9.1.18) 8665474 PMC172879

[7] Green DE, Converse KA, Schrader AK. 2002 Epizootiology of sixty-four amphibian morbidity and mortality events in the USA. Ann. N.Y. Acad. Sci. **969**, 323-339. (10.1111/j.1749-6632.2002.tb04400.x) 12381613

[8] Landsberg JH, Kiryu Y, Tabuchi M, Waltzek TB, Enge KM, Reintjes-Tolen S, Preston A, Pessier AP. 2013 Co-infection by alveolate parasites and frog virus 3-like ranavirus during an amphibian larval mortality event in Florida, USA. Dis. Aquat. Organ. **105**, 89-99. (10.3354/dao02625) 23872853

[9] Green DE, Feldman SH, Wimsatt J. 2003 Emergence of a *Perkinsus*-like agent in anuran liver during die-offs of local populations: PCR detection and phylogenetic characterization. Proc. Am. Assoc. Zoo. Vet. **2003**, 120-121.

[10] Burreson EM. 2008 Misuse of PCR assay for diagnosis of mollusc protistan infections. Dis. Aquat. Organ. **80**, 81-83. (10.3354/dao01925) 18714688

[11] Smilansky V, Chambouvet A, Reeves M, Richards TA, Milner DS. 2021 A novel duplex qPCR assay for stepwise detection of multiple Perkinsea protistan infections of amphibian tissues. R. Soc. Open Sci. **8**, 202150. (10.1098/rsos.202150) 33959367 PMC8074936

[12] Gouy M, Guindon S, Gascuel O. 2010 SeaView version 4: a multiplatform graphical user interface for sequence alignment and phylogenetic tree building. Mol. Biol. Evol. **27**, 221-224. (10.1093/molbev/msp259) 19854763

[13] Smilansky V. 2020 Smilansky Perkinsea tadpole MSAs. Dataset. *Figshare*. (10.6084/m9.figshare.13108136.v1)

[14] Lockhart PJ, Steel MA, Hendy MD, Penny D. 1994 Recovering evolutionary trees under a more realistic model of sequence evolution. Mol. Biol. Evol. **11**, 605-612. (10.1093/oxfordjournals.molbev.a040136) 19391266

[15] Gascuel O. 1997 BIONJ: an improved version of the NJ algorithm based on a simple model of sequence data. Mol. Biol. Evol. **14**, 685-695. (10.1093/oxfordjournals.molbev.a025808) 9254330

[16] Foster PG, Hickey DA. 1999 Compositional bias may affect both DNA-based and protein-based phylogenetic reconstructions. J. Mol. Evol. **48**, 284-290. (10.1007/PL00006471) 10093217

[17] Nguyen LT, Schmidt HA, von Haeseler A, Minh BQ. 2015 IQ-TREE: a fast and effective stochastic algorithm for estimating maximum-likelihood phylogenies. Mol. Biol. Evol. **32**, 268-274. (10.1093/molbev/msu300) 25371430 PMC4271533

[18] Kalyaanamoorthy S, Minh BQ, Wong TKF, von Haeseler A, Jermiin LS. 2017 ModelFinder: fast model selection for accurate phylogenetic estimates. Nat. Methods **14**, 587-589. (10.1038/nmeth.4285) 28481363 PMC5453245

[19] Pomerantz A, Peñafiel N, Arteaga A, Bustamante L, Pichardo F, Coloma LA, Barrio-Amorós CL, Salazar-Valenzuela D, Prost S. 2018 Real-time DNA barcoding in a rainforest using nanopore sequencing: opportunities for rapid biodiversity assessments and local capacity building. Gigascience **7**, giy033. (10.1093/gigascience/giy033) PMC5905381 29617771

[20] Lips KR et al. 2006 Emerging infectious disease and the loss of biodiversity in a Neotropical amphibian community. Proc. Natl Acad. Sci. USA **103**, 3165. (10.1073/pnas.0506889103) 16481617 PMC1413869

[21] Pramuk JB, Robertson T, Sites Jr JW, Noonan BP. 2008 Around the world in 10 million years: biogeography of the nearly cosmopolitan true toads (Anura: Bufonidae). Glob. Ecol. Biogeogr. **17**, 72-83. (10.1111/j.1466-8238.2007.00348.x)

[22] McCutchan TF, de la Cruz VF, Lal AA, Gunderson JH, Elwood HJ, Sogin ML. 1988 Primary sequences of two small subunit ribosomal RNA genes from *Plasmodium falciparum*. Mol. Biochem. Parasitol. **28**, 63-68. (10.1016/0166-6851(88)90181-8) 2836731

[23] Pagenkopp Lohan KM, Hill-Spanik KM, Torchin ME, Fleischer RC, Carnegie RB, Reece KS, Ruiz GM. 2018 Phylogeography and connectivity of molluscan parasites: *Perkinsus* spp. in Panama and beyond. Int. J. Parasitol. **48**, 135-144. (10.1016/j.ijpara.2017.08.014) 29108906

[24] Vilas R, Cao A, Pardo BG, Fernández S, Villalba A, Martínez P. 2011 Very low microsatellite polymorphism and large heterozygote deficits suggest founder effects and cryptic structure in the parasite *Perkinsus olseni*. Infect. Genet. Evol. **11**, 904-114. (10.1016/j.meegid.2011.02.015) 21376141

[25] Whitfield SM, Lips KR, Donnelly MA. 2016 Amphibian decline and conservation in Central America. Copeia **104**, 351-379. (10.1643/CH-15-300)

[26] Peeler EJ, Oidtmann BC, Midtlyng PJ, Miossec L, Gozlan RE. 2011 Non-native aquatic animals introductions have driven disease emergence in Europe. Biol. Invasions **13**, 1291-1303. (10.1007/s10530-010-9890-9)

[27] Naylor RL, Williams SL, Strong DR. 2001 Aquaculture—a gateway for exotic species. Science **294**, 1655. (10.1126/science.1064875) 11721035

[28] Picco AM, Collins JP. 2008 Amphibian commerce as a likely source of pathogen pollution. Conserv. Biol. **22**, 1582-1589. (10.1111/j.1523-1739.2008.01025.x) 18717688

[29] McKenzie VJ, Peterson AC. 2012 Pathogen pollution and the emergence of a deadly amphibian pathogen. Mol. Ecol. **21**, 5151-5154. (10.1111/mec.12013) 23075064

[30] Stuart SN, Chanson JS, Cox NA, Young BE, Rodrigues ASL, Fischman DL, Waller RW. 2004 Status and trends of amphibian declines and extinctions worldwide. Science **306**, 1783. (10.1126/science.1103538) 15486254

[31] IUCN CI, and NatureServe. An analysis of amphibians on the 2008 IUCN Red List. See www.iucn-amphibians.org/red-listing/global-amphibian-assessment (accessed 02/01/2018).

[32] Lewis CHR, Richards-Zawacki CL, Ibáñez R, Luedtke J, Voyles J, Houser P, Gratwicke B. 2019 Conserving Panamanian harlequin frogs by integrating captive-breeding and research programs. Biol. Conserv. **236**, 180-187. (10.1016/j.biocon.2019.05.029)

[33] Griffiths RA, Pavajeau L. 2008 Captive breeding, reintroduction, and the conservation of amphibians. Conserv. Biol. **22**, 852-861. (10.1111/j.1523-1739.2008.00967.x) 18616746

[34] Smilansky V, Jirků M, Milner DS, Ibáñez R, Gratwicke B, Nicholls A, Lukeš J, Chambouvet A, Richards TA. 2021 Expanded host and geographic range of tadpole associations with the Severe Perkinsea Infection group. *Figshare*. (10.6084/m9.figshare.c.5448491) PMC8205526 34129800

